# Identification of TIMP1 as a Key Regulator of Ferroptosis in Ulcerative Colitis Through Bioinformatics and Functional Experiments

**DOI:** 10.1155/grp/6894499

**Published:** 2026-08-12

**Authors:** Xuan Zhou, Qiao Qiao, Jie Yang, Huiming Tu

**Affiliations:** ^1^ Department of Gastroenterology, Affiliated Hospital of Jiangnan University, Wuxi, China, jiangnan.edu.cn

**Keywords:** ferroptosis, intestinal epithelial permeability, PGRMC1, TIMP1, ulcerative colitis

## Abstract

**Background:**

Ferroptosis is an iron‐dependent form of regulated cell death, whereas the precise mechanism of ferroptosis in the pathogenesis of ulcerative colitis (UC) remains to be elucidated. The tissue inhibitor Metalloproteinase 1 (TIMP1) is involved in ferroptosis of UC, whereas its regulatory function remains unclear. This study is aimed at exploring the potential effect on ferroptosis in UC.

**Methods:**

Gene expression profiling data of colonic biopsies from UC patients and healthy controls were acquired from the GSE87466 dataset. The expression of TIMP1, GPX4, and SLC7A11 was determined using western blot. The mRNA levels of TIMP1, PGRMC1, IL‐1*β*, and IL‐6 were measured using reverse transcription quantitative PCR (RT‐qPCR). The Fe^2+^ and malondialdehyde (MDA) contents were detected with commercial kits. The reactive oxygen species (ROS) production was analyzed via flow cytometry. The integrity of the intestinal epithelium was evaluated using a fluorescein isothiocyanate (FITC)–dextran permeability assay and transepithelial electrical resistance (TEER) measurements.

**Results:**

Bioinformatics identified TIMP1 and PGRMC1 as core genes related to ferroptosis of UC. Both TIMP1 and PGRMC1 were upregulated in colonic biopsies from UC patients in the GSE87466 dataset. The mRNA expression was induced after LPS treatment, whereas PGRMC1 was not significantly altered. TIMP1 knockdown inhibited the expression of IL‐1*β* and IL‐6; reduced Fe^2+^, MDA, and ROS levels; and upregulated the expression of GPX4 and SLC7A11 in LPS‐induced intestinal epithelial cells. Furthermore, TIMP1 knockdown reduced the intestinal epithelial leakage in vitro.

**Conclusion:**

TIMP1 regulated ferroptosis, inflammatory response, and intestinal epithelial permeability in LPS‐induced intestinal epithelial cells, suggesting TIMP1 as a potential therapeutic target for UC.

## 1. Introduction

Ulcerative colitis (UC) is a generic, chronic, recurrent inflammatory condition mostly affecting the mucosa and submucosa of the colorectum [1, 2]. The incidence and prevalence of UC have had a consistent increase in recent years [3]. The ailment has a prolonged duration and a diverse array of lesions [4]. Moreover, it is crucial to acknowledge that the primary clinical manifestations associated with this condition include recurrent diarrhea, severe abdominal pain, viscous mucus discharge, visibly blood‐stained feces, and other debilitating symptoms that significantly impair an individual′s quality of life [5]. Notwithstanding the availability of numerous treatments, over 15% of individuals with UC ultimately necessitate surgical intervention owing to the ineffectiveness of medicinal therapy [6, 7]. The expenses related to surgical complications impose a significant economic burden. The pathogenesis of UC is obscure and intricate. Evidence is increasingly indicating a close relationship between oxidative stress and inflammation, influenced by genetics, gut flora, the host immune system, and environmental variables [8–11]. Research examining the molecules underlying the pathophysiology of UC is crucial.

In recent years, ferroptosis, a distinct form of cell death characterized by iron accumulation and lipid peroxidation, has been identified as a crucial factor in the onset and advancement of various intestinal disorders, including intestinal ischemia/reperfusion injury, inflammatory bowel disease (IBD), and colorectal cancer (CRC) [12–14]. Ferroptosis is governed by various cellular metabolic pathways, encompassing redox homeostasis, iron metabolism, mitochondrial function, amino acid metabolism, and lipid metabolism [15, 16]. An increasing number of studies have identified ferroptosis in the colonic tissues of UC patients and animal models of the condition [13, 17, 18]. The principal events of ferroptosis are intricately linked to the pathogenic mechanisms of UC, encompassing glutathione depletion, GPX4 inactivation, lipid peroxidation, iron homeostasis disruption, and lipoxygenase overexpression [12, 19]. Ferroptosis contributes to persistent inflammation in UC by inducing deadly reactive oxygen species (ROS) buildup, iron overload, and unregulated lipid peroxidation, leading to the demise of intestinal epithelial cells (IECs) [18]. Notwithstanding these findings, the significance and mechanisms of ferroptosis in UC remain ambiguous and necessitate additional investigation.

Tissue inhibitor Metalloproteinase 1 (TIMP1), sometimes referred to as EPA, is a member of a metalloproteinase family that prevents the degradation of the extracellular matrix [20]. TIMP1 has been documented in numerous conditions, including subarachnoid hemorrhage, different malignancies, ischemic stroke, Type 2 diabetic osteoporosis, and UC [21–26]. In individuals with IBD, TIMP1 expression is markedly increased in colonic tissue and serum, corresponding with the severity of the disease [27]. Moreover, bioinformatics has recognized TIMP1 in the context of ferroptosis. In Type 2 diabetic osteoporosis, the knockdown of TIMP1 suppresses ferroptosis in osteoblasts triggered by elevated glucose and lipid levels [23]. Zhang et al. [28] demonstrate that TIMP1 is identified as one of the four ferroptosis‐related genes (TIMP1, LPIN1, SOCS1, and CD44) possessing diagnostic significance for UC. The expression of the four genes is elevated in dextran sulfate sodium (DSS) salt–induced mice colitis and correlates with several immune cells, including neutrophils, M0 and M1 macrophages, B cells, and NK cells [28]. Moreover, TIMP1 is a principal target via which oxymatrine regulates ferroptosis and inflammation in UC [29]. The treatment of oxymatrine inhibits the expression of key genes, such as TIMP1, and mitigates the pathological damage linked to UC by enhancing ferroptosis and inflammatory responses in colonic tissue [29]. Although numerous studies have recognized the role of TIMP1 in the ferroptosis associated with UC, limited research has investigated the precise function of TIMP1 at the single‐gene level in this context.

This study linked the UC dataset from the GEO database with ferroptosis‐related genes from FerrDB for bioinformatics analysis to discover possible biomarkers. TIMP1 was recognized as the principal gene associated with ferroptosis in UC and showed upregulation in samples from UC patients in public databases and lipopolysaccharide (LPS)‐induced IECs. The impact of TIMP1 knockdown on ferroptosis, inflammation, and intestinal epithelial permeability was investigated, clarifying the function of TIMP1 in the ferroptosis associated with UC.

## 2. Materials and Methods

### 2.1. Data Acquisition

The datasets GSE87466 and GSE47908 were acquired from the GEO public database (http://www.ncbi.nlm.nih.gov/geo), comprising colonic biopsies from 87 UC patients and 21 healthy controls to 45 UC patients and 15 healthy controls, respectively. Subsequent to the gene reannotation of the dataset probes via platform files, the data underwent log transformation and normalization. Two hundred sixty‐four ferroptosis‐related genes were obtained from the FerrDB database (http://www.zhounan.org/ferrdb/current/).

### 2.2. Weighted Gene Coexpression Network Analysis (WGCNA)

WGCNA was conducted on the GSE87466 dataset using the “WGCNA” tool in R software to identify gene modules. Initially, hierarchical clustering of the samples was conducted. Utilizing the correlation coefficient R^2^ = 0.86, the “picksoftthreshold” function was employed to develop the gene expression patterns of scale‐free networks. Following the acquisition of the modules, the first principal component′s module, derived from various module eigengenes (ME), was correlated with the clinical characteristics of the evaluation module‐trait correlations. The modules exhibiting the most pronounced positive and negative correlations in the module‐trait associations were subsequently identified.

### 2.3. Gene Function Enrichment Analysis

An enrichment analysis of the 18 intersecting genes was conducted utilizing the online Sangerbox platform, focusing on the Gene Ontology (GO) and Kyoto Encyclopedia of Genes and Genomes (KEGG). Pathways with adjusted *p* < 0.05 were deemed significant. The 10 most significant enrichment findings were ultimately visualized.

### 2.4. Machine Learning

Three machine learning algorithms, including LASSO regression, support vector machine recursive feature elimination (SVM‐RFE), and Random Forest (RF), were utilized to find possible biomarkers from the 18 overlapping genes revealed after WGCNA. LASSO regression analysis was conducted utilizing the “glmnet” package in R. Subsequently, five cross‐validations were conducted to ascertain the optimal value for *λ*. This work identifies four important hub genes linked to ferroptosis in UC, identified by the *λ* using the minimal criterion. The R package “e1071 1.7.16” was utilized to execute the SVM‐RFE method, employing k‐fold cross‐validation with k set to five and a threshold of 100. Genes with the best accuracy or minimal error were chosen for further study. The RF technique was implemented with the R package “randomForest 4.7.1.2.” RF models were trained across five folds for each quantity of trees, and the mean error rate on the test set was computed. The model′s performance was assessed using 5‐fold cross‐validation. The cross‐validation findings indicated the optimal number of trees that produced the minimal average error rate, along with the relevant genes identified by the model.

### 2.5. Cell Culture and Treatment

The human normal colon epithelial cell line (NCM460) was obtained from SUNNCELL (Wuhan, China). The cells were validated using short tandem repeat profiling and verified to be devoid of mycoplasma infection. All cells were cultured in RPMI‐1640 media enriched with 10% fetal bovine serum in a humidified incubator at 37°C with 5% CO_2_. Cells were subjected to 10‐*μg*/mL LPS (sigma) for 24 h postplating to elicit cellular inflammation.

### 2.6. Cell Transfection

The lentiviral particles containing shRNA directed against TIMP1 (shTIMP1) or the matching control (shNC) were procured from Genechem (Shanghai, China). NCM460 cells were transduced with a lentivirus‐containing media supplemented with 12‐*μg*/mL polybrene (Sigma–Aldrich) for 24 h. Stable cell lines were established using selection with 1.5‐*μg*/mL puromycin (Sigma–Aldrich) over a period of 14 days.

### 2.7. Quantification of Malondialdehyde (MDA), Fe^2+^, and ROS

NCM460 cells transfected with shTIMP1, shNC, or without transfection were incubated in six‐well plates overnight. The cells were subjected to treatment with or without 10‐*μg*/mL LPS. Adherent cells were subjected to the lysis buffer for the detection of MDA and Fe^2+^. The supernatant was obtained following centrifugation. The levels of MDA and Fe^2+^ in the cell lysates were measured using the Lipid Peroxidation MDA Assay Kit (Beyotime, Shanghai, China) and the Cellular Ferrous Iron Content Detection Kit (Yeasen, Shanghai, China), respectively. The acquired levels were standardized to total protein concentration as determined by the BCA test. To quantify ROS, cells were trypsinized and treated with 10 *μ*M BODIPY 581/591 C11 (Jiancheng Bioengineering Institute, Nanjing, China) for 30 min at 37°C, thereafter analyzed using a flow cytometer.

### 2.8. Reverse Transcription Quantitative PCR (RT‐qPCR)

Total RNA was extracted with a total RNA isolation kit (Takara, Japan). After quantifying RNA with the NanoDrop 2000, 1 *μg* of total RNA was reverse‐transcribed into cDNA using HiScript RT SuperMix for qPCR (Vazyme). SYBR Green‐based quantitative PCR amplification was performed utilizing SYBR Green Master Mix (Vazyme). Expression levels normalized to *β*‐actin were determined using the 2^−*ΔΔ*Ct^ method. The primers were presented in Table 1.

**Table 1:** Primers for RT‐qPCR.

Name		Primers for PCR (5 ^′^‐3 ^′^)
TIMP1	Forward	GCGGATACTTCCACAGGTCC
	Reverse	GTAGGTCTTGGTGAAGCCCC
PGRMC1	Forward	GGGCCCGTCAAGTATCATCA
	Reverse	TCCGGGCACTCTCATCTTTT
IL‐6	Forward	CCACCGGGAACGAAAGAGAA
	Reverse	GAGAAGGCAACTGGACCGAA
IL‐1*β*	Forward	AACCTCTTCGAGGCACAAGG
	Reverse	AGCCATCATTTCACTGGCGA
*β*‐actin	Forward	CTTCGCGGGCGACGAT
	Reverse	CCACATAGGAATCCTTCTGACC

#### 2.8.1. Receiver Operating Characteristic (ROC) Assessment

The ROC analysis was performed using the “pROC” package to evaluate its diagnostic ability in UC.

### 2.9. Western Blot

Cells were lysed with precooled RIPA buffer (Solarbio, Beijing, China), and protein quantities were quantified using the BCA technique. Proteins were applied in identical quantities onto 12% SDS‐PAGE. Following transfer to nitrocellulose membranes, they were subjected to blocking with 5% nonfat dairy milk. Membranes were subsequently incubated with primary antibodies overnight at 4°C, including anti‐TIMP1 (16644‐1‐AP, 1:1000, Proteintech, Wuhan, China), anti‐GPX4 (67763‐1‐Ig, 1:1000, Proteintech), anti‐SLC7A11 (26864‐1‐AP, 1:1000, Proteintech), and anti‐*β*‐actin (66009‐1‐Ig, 1:20000, Proteintech). On the following day, the membranes were incubated with secondary HRP‐conjugated antibodies at 37°C for 1 h. Ultimately, membrane blots were generated utilizing an ECL reagent (NCM Biotech, Suzhou, China). Protein concentrations were measured via the ImageJ program.

### 2.10. Assessment of Intestinal Permeability

The integrity of the intestinal epithelium was evaluated using a fluorescein isothiocyanate (FITC)–dextran permeability assay and transepithelial electrical resistance (TEER) measurements [30]. The NCM460 cells, transfected with shTIMP1, shNC, or without transfection, were grown to 70% confluence, harvested, and adjusted to approximately 2 × 10^5^ cells/mL. One milliliter of cell suspension was deposited onto collagen‐coated polycarbonate permeable support membranes with 0.4 mm pores (Corning, United States). The medium was changed every 2 days. The TEER of both blank membranes and membranes containing NCM460 cell layers was assessed using the Millicell‐ERS2 (Merck–Millipore) apparatus. TEER readings were generally expressed in units of *Ω* cm^2^ and computed as follows: TEER value = (mean resistance of each well − mean resistance of blank) × membrane area. The epithelial permeability of NCM460 cells was evaluated by measuring the flux of FITC‐dextran (Sigma–Aldrich). FITC‐dextran was introduced at a concentration of 5 *μ*M into the apical chamber with 100 *μ*L of PBS, whereas the basolateral chamber held 500 *μ*L of PBS. Following a 6‐h culture period, 100 *μ*L of the basolateral media was collected and analyzed using a microplate reader. The measured fluorescence intensity was standardized against the control.

### 2.11. Statistical Analysis

Statistical analyses were conducted utilizing GraphPad Prism v8.0.1 software. The variables were presented as mean ± standard deviation (SD). Two‐tailed Student′s *t*‐tests or one‐way ANOVA with Tukey′s post hoc analysis were utilized for group comparisons. Statistical significance was established at *p* < 0.05.

## 3. Results

### 3.1. Construction of Coexpression Network and Identification of UC Core Genes

**Figure 1:**
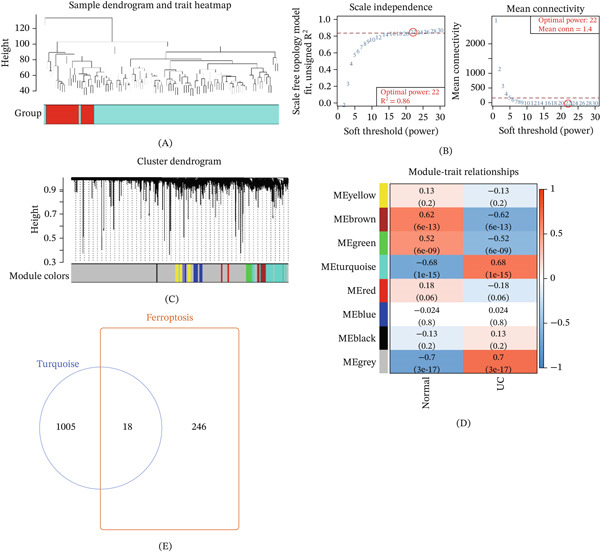
Construction of the coexpression network. (A) The sample dendrogram and feature heat map were drawn using the average clustering method for hierarchical clustering of samples, with each branch representing a sample. (B) Soft threshold (power = 22) and scale‐free topology fit index (R^2^ = 0.86). (C) Gene hierarchy tree‐clustering diagram. The graph indicated different genes horizontally and the uncorrelatedness between genes vertically. The lower the branch, the less uncorrelated the genes within the branch. (D) Heatmap illustrating the correlations between the module and ulcerative colitis (UC) features. The values in the small cells of the graph represented the two calculated correlation coefficients between the trait and each module, as well as the corresponding *p* values. (E) Venn diagram of the intersection of turquoise module genes and ferroptosis‐related genes.

The dataset GSE87466 (platform file GPL13158) was downloaded from the GEO public database, which contained gene expression data of colon biopsies from 87 UC patients and 21 healthy control subjects. A gene coexpression network using the WGCNA algorithm was constructed to precisely excavate the central genes associated with the UC phenotype. The sample hierarchical cluster analysis results showed good clustering among the samples, with no significant outliers (Figure 1A). The soft threshold was set to 22 to satisfy the scale‐free topology of the network, where the corresponding *R* ^2^ was 0.86, and the average connectivity was 1.4 (Figure 1B). A gene hierarchy clustering dendrogram was constructed by gene correlation, and a total of eight gene modules were identified (Figure 1C). Figure 1D displayed the correlation coefficient and *p* values of each gene module. Subsequently, the “turquoise” module, containing 1023 genes, was identified as the most clinically valuable module in UC (Cor = 0.68, *p* = 1 × 10^−15^) based on the correlation of module feature value with UC phenotypes. Two hundred sixty‐four ferroptosis‐related genes were obtained from the FerrDB (http://www.zhounan.org/ferrdb/current/) database for further analysis. By intersecting the genes in the turquoise module and ferroptosis‐related genes, a total of 18 core genes were identified (Figure 1E).

### 3.2. Enrichment Analysis

**Figure 2:**
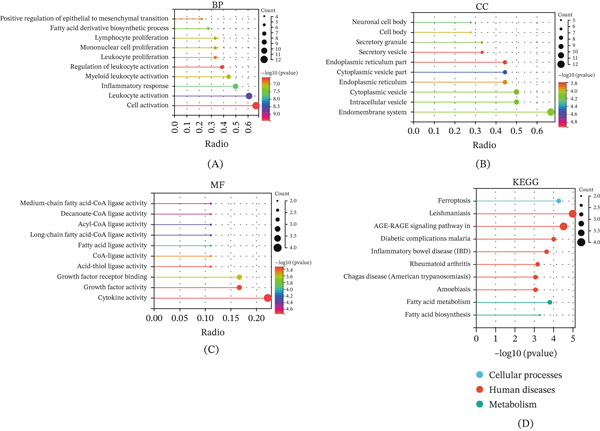
Functional enrichment analysis. GO enrichment results of the (A) BP, (B) CC, and (C) MF analysis. (D) Functional annotation of the KEGG signaling pathway of signature genes. Abbreviations: GO, Gene Ontology; KEGG, Kyoto Encyclopedia of Genes and Genomes.

Subsequently, functional enrichment analysis of the 18 core genes was performed to explore the biological functions and potential pathways associated with UC. The GO annotation results showed that these genes were enriched in the following biological processes (BPs), such as positive regulation of epithelial to mesenchymal transition, fatty acid derivative biosynthetic process, lymphocyte proliferation, mononuclear cell proliferation, and leukocyte proliferation (Figure 2A). The results of CC contained neuronal cell body, secretory granule, secretory vesicle, and endoplasmic reticulum part (Figure 2B). The enrichment results of MF involved medium‐chain fatty acid‐CoA ligase activity, decanoate‐CoA ligase activity, acyl‐CoA ligase activity, long‐chain fatty acid‐CoA ligase activity, and fatty acid ligase activity (Figure 2C). The results of the KEGG signaling pathway analysis demonstrated that the signature genes were most enriched in ferroptosis, IBD, rheumatoid arthritis, and fatty acid metabolism (Figure 2D). These results revealed that the core genes were significantly enriched in ferroptosis.

### 3.3. Machine Learning

**Figure 3:**
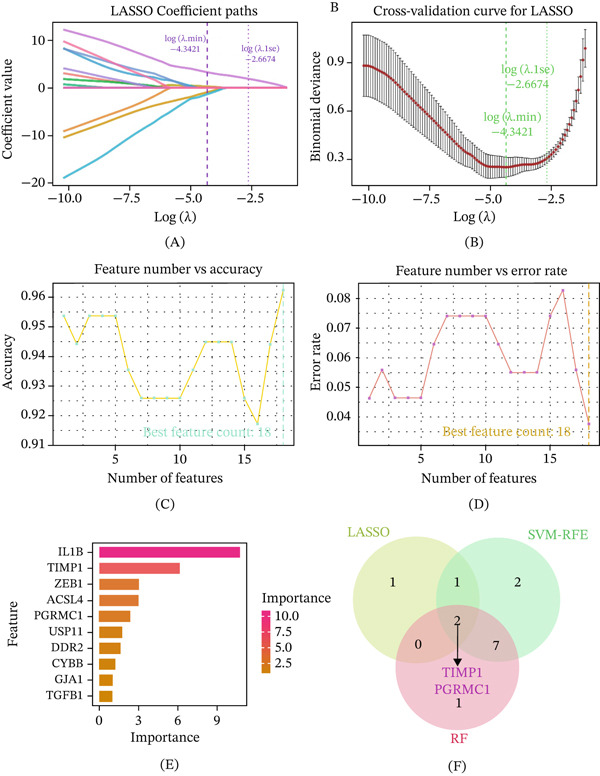
Machine learning was used to screen key genes for ferroptosis of UC. (A and B) Biomarker screening in LASSO‐cox models. The number of genes corresponding to the lowest point of the curve was most suitable for assessing key genes. (C and D) Screening for biomarkers via the SVM‐RFE algorithm. (E) The random forest method was used to screen biomarkers, sequencing the importance of genes according to MeanDecreaseGini values. (F) Venn diagram of the intersection of genes from three algorithms.

Several machine learning algorithms were further applied to figure out core genes. As shown in Figure 3A,B, the LASSO algorithm identified four potential biomarkers. SVM‐RFE algorithm revealed that a model involving 18 genes achieved the lowest Root Mean Square Error (RMSE) (Figure 3C,D). Moreover, 10 genes were identified via the RF algorithm (Figure 3E). By intersecting the results of these three algorithms, TIMP1 and progesterone receptor membrane component‐1 (PGRMC1) were defined as the common characteristic genes in ferroptosis of UC (Figure 3F).

### 3.4. TIMP1 Was Involved in the Ferroptosis of UC

**Figure 4:**
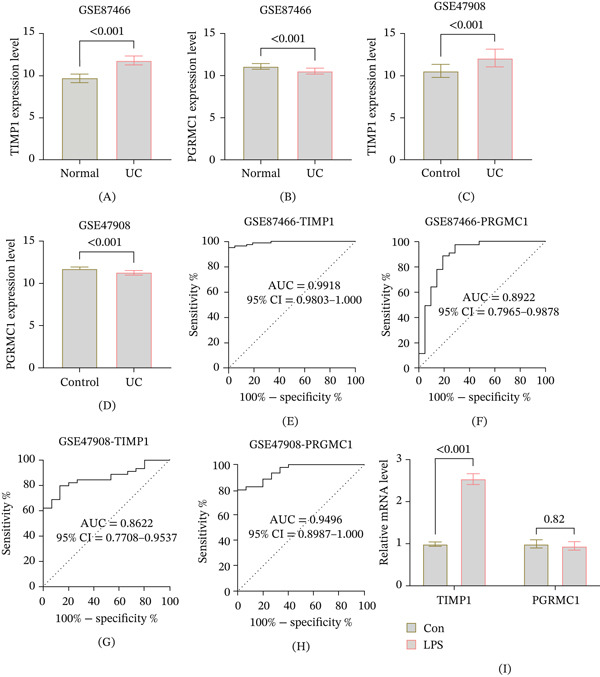
The expression of TIMP1 and PGRMC1. (A and B) The distribution of TIMP1 and PGRMC1 expression between UC patients and healthy controls from GSE87466 dataset. (C and D) The distribution of TIMP1 and PGRMC1 expression between UC patients and healthy controls from GSE47908 dataset. (E–H) The diagnostic value of TIMP1 and PGRMC1 for UC in GSE87466 and GSE47908 datasets was assessed using ROC analysis. (I) The mRNA expression of PGRMC1 and TIMP1 in NCM460 cells treated with or without 10‐*μg*/mL LPS was measured using RT‐qPCR.

To further explore the function of the identified genes in UC, the expression of the two genes was first investigated. The mRNA expression of PGRMC1 and TIMP1 was analyzed in public datasets and LPS‐induced cells. Compared with healthy controls, PGRMC1 expression was downregulated, whereas TIMP1 expression was upregulated in UC patients from the GSE87466 dataset (both *p* < 0.001, Figure 4A,B). The expression of these two genes showed similar results in UC patients from the GSE47908 dataset, which contained gene expression data of colon biopsies from 45 UC patients and 15 healthy control subjects (Figure 4C,D). In addition, the study also explored the diagnostic value of PGRMC1 and TIMP1 for UC. The ROC analysis showed that in the GSE87466 dataset, the areas under the curve (AUCs) for TIMP1 and PGRMC1 were 0.9918 (95% CI: 0.9803–1.000) and 0.8922 (95% CI: 0.7965–0.9878), whereas those in the GSE47908 dataset were 0.8622 (95% CI: 0.7708–0.9537) and 0.9496 (95% CI: 0.8987–1.000), respectively (Figure 4E–H), indicating that both TIMP1 and PGRMC1 demonstrated strong diagnostic potential for UC in both datasets. The mRNA expression of PGRMC1 and TIMP1 was further measured in LPS‐induced NCM460 cells. Compared with cells without LPS treatment, TIMP1 was upregulated in LPS‐treated cells (*p* < 0.001), whereas PGRMC1 expression was mildly downregulated upon LPS treatment (*p* = 0.82, Figure 4I). Therefore, TIMP1 was selected as the target gene in subsequent experiments for its upregulation in UC patients and LPS‐induced NCM460 cells.

### 3.5. TIMP1 Knockdown Alleviated Ferroptosis in Inflamed Colonic Epithelial Cells

**Figure 5:**
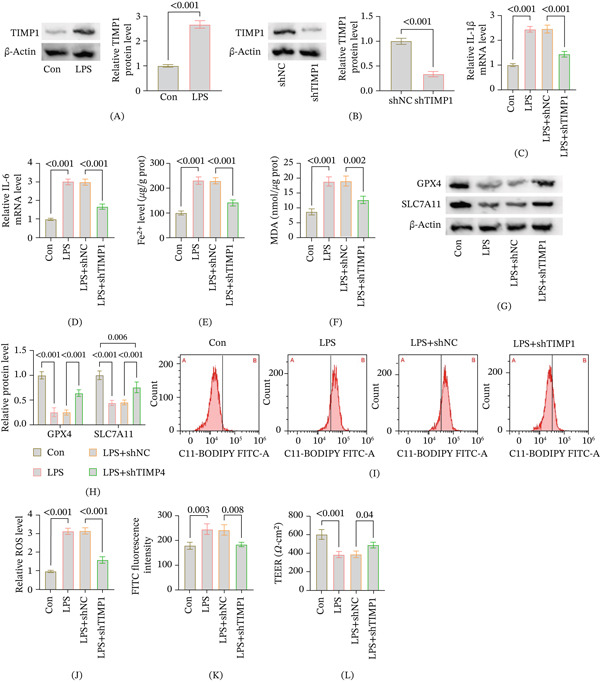
The role of TIMP1 in LPS‐induced NCM460 cells. (A) The expression of TIMP1 in NCM460 cells treated with or without 10‐*μg*/mL LPS was measured using western blot. (B) The expression of TIMP1 in NCM460 cells transfected with shNC or shTIMP1 was measured using western blot. (C and D) NCM460 cells were treated with LPS (10 *μg*/mL), LPS (10 *μ* *g*/mL) + shNC, LPS (10 *μ* *g*/mL) + shTIMP1, or without (control). The IL‐1*β* and IL‐6 levels in treated cells were detected using RT‐qPCR. (E and F) The levels of Fe^2+^ and MDA in treated cells were analyzed using commercial kits. (G and H) The protein expression of GPX4 and SLC7A11 in treated cells was measured using western blot. (I) The ROS production in treated cells was analyzed using flow cytometry. (K and L) Assessment of intestinal epithelial cell integrity via FITC‐dextran permeability assay and TEER.

To explore the potential association between TIMP1 and ferroptosis in UC, NCM460 cells were subjected to LPS stimulation to establish inflammatory colonic epithelial models. The protein level of TIMP1 was induced in inflamed colonic epithelial cells (Figure 5A). NCM460 cells were transfected with shNC or shTIMP1 for further functional exploration. The downregulation of TIMP1 in treated cells was confirmed via western blot, indicating the successful silencing of TIMP1 (Figure 5B). Transfected NCM460 cells were treated with LPS (10 *μg*/mL) or without (control). The results showed that the inflammatory cytokines IL‐1*β* and IL‐6 in TIMP1 knockdown colon epithelial cells were lower than those in the control group (Figure 5C,D). Iron content assay presented significantly higher Fe^2+^ content in NCM460 cells with LPS induction, and lower Fe^2+^ content in TIMP1 silencing cells compared to the control group (Figure 5E). Lipid peroxidation levels were assessed by MDA assay. Under LPS intervention, the MDA level of NCM460 cells was significantly increased, whereas TIMP1 downregulation decreased the MDA level (Figure 5F). Western blot analysis showed that in inflammatory colonic epithelial cells, the expression of ferroptosis‐protective molecules (GPX4 and SLC7A11) was upregulated. Moreover, the expressions of GPX4 and SLC7A11 were decreased after TIMP1 knockdown (Figure 5G,H). C11‐BODIPY flow cytometry showed increased ROS production in LPS‐induced cells, which was impaired by TIMP1 silencing (Figure 5I,J). The intestinal injury was also investigated using FITC‐dextran and TEER. The results indicated that, in contrast to the untransfected cells or cells transfected with shNC in the monolayer, a discernible reduction in intestinal epithelial leakage was observed in the cells transfected with TIMP1 knockdown (Figure 5K,L). Taken together, the above results confirmed that ferroptosis occurred in LPS‐induced NCM460 cells and that TIMP1 knockdown suppressed ferroptosis and inflammation in human colonic epithelial cells.

## 4. Discussion

UC denotes an inflammatory condition marked by ongoing injury to colonic epithelial cells [2]. The pathophysiology of UC remains ambiguous. Current research indicates that ferroptosis is linked to the advancement of UC [12]. This work identified and verified TIMP1 as the key target of ferroptosis in UC.

Ferroptosis is recognized as an iron‐dependent, controlled form of cell death, separate from other processes of cell death [16]. Recent studies indicate that ferroptosis is linked to intestinal disorders. The suppression of ferroptosis is anticipated to be a promising treatment approach for UC [31–33]. Deletion of epithelial Piezo1 mitigates intestinal barrier damage by decreasing lipid peroxidation, improving mitochondrial function, and modifying the expression of ferroptosis‐related proteins in UC [34]. In the DSS‐induced colitis animal model, the overexpression of HMGB1 was associated with alterations in TLR4, NF‐*κ*B, and GPX4 expression, as well as the upregulation of ferroptosis‐related genes [35]. Conversely, the inhibition of HMGB1 mitigated inflammation, restored barrier function, and reversed ferroptosis [35]. This study utilized bioinformatics to identify PGRMC1 and TIMP1 as key genes associated with ferroptosis in UC. Both genes exhibited excellent diagnostic performance for UC. PGRMC1 is a recognized hormone receptor that interacts with intracellular free iron, therefore decreasing its content and preventing ferroptosis in triple‐negative breast cancer [36, 37]. The research indicated that PGRMC1 expression was elevated in colonic samples from UC patients in a public dataset, although LPS treatment did not significantly influence its expression in IECs. The expression of TIMP1 was elevated in colonic samples from UC patients and in LPS‐induced IECs. Moreover, TIMP1 silencing diminished the expression of inflammatory cytokines, Fe^2+^ levels, and lipid peroxidation while enhancing the production of ferroptosis‐protective molecules (GPX4 and SLC7A11) and preserving intestinal epithelial integrity. Comparable findings have been observed in CRC, where TIMP1 knockdown increases sensitivity to ferroptosis, inhibits CRC cell proliferation and migration, and diminishes the expression of ferroptosis regulators GPX4 and SLC7A11 [38]. Numerous pathogenic pathways have been identified as contributing to ferroptosis in UC [12, 35, 39]. What are the underlying processes by which TIMP1 regulates ferroptosis in UC? The transferrin receptor (TFRC) is a crucial iron acquisition channel in the ferroptosis mechanism [40]. TIMP1 can impede the ubiquitination of TFRC, enhancing its stability, which subsequently elevates intracellular Fe^2+^ levels, fosters the formation of ROS and lipid peroxidation, and triggers ferroptosis in osteoblasts [23]. This research focuses on osteoblasts, suggesting that TIMP1 may also modulate ferroptosis in UC through TFRC.

The pathophysiology of UC is intricate, with gut barrier impairment significantly contributing to disease advancement [41]. The epithelial cell layer constitutes the primary element of the intestinal barrier, establishing a mechanical and metabolic separation between luminal bacteria and mucosal immune cells [42]. The natural demise of IECs is advantageous for preserving their functionality, ensuring ongoing renewal capability, and upholding tissue homeostasis. Excessive IEC mortality leads to heightened intestinal permeability and malfunction of the intestinal mucosal barrier [43]. Enhanced intestinal permeability was noted in LPS‐induced IECs, but TIMP1 knockdown ameliorated the integrity of the epithelial cell layer. A mouse experiment shows that TIMP1 regulates endothelial barrier integrity by interacting with the CD63/integrin *β*1 complex in traumatic brain injury [44]. Besides, TIMP1 inhibits MMPs, preventing the excessive degradation of tight junction proteins, therefore preserving TEER and decreasing permeability [45]. Excessive MMP activity or disturbed TIMP1 control may result in compromised barrier function [45]. TIMP1 plays a role in regulating inflammation and intestinal fibrosis in chronic enteritis [45]. The progression of fibrosis modifies the mechanical tension and milieu of the intestinal wall, thereby impacting the integrity of epithelial cells indirectly.

The current study has some limitations. The bioinformatics analysis in this study utilized public databases. Although the expression of TIMP1 has been verified in cellular models, additional experiments in colonic tissues from individuals with clinical UC are necessary for a more comprehensive interpretation of the results. Secondly, the study was confined to IECs; future research should focus on developing mouse models that better align with the characteristics of UC. Third, although the study concentrated on the function of TIMP1 in IECs, it did not investigate the underlying mechanisms. The involvement of ferroptosis in TIMP1‐mediated effects in UC should also be confirmed using ferroptosis‐specific inhibitors (such as ferrostatin‐1) or inducers (such as erastin) in future studies.

## 5. Conclusion

In summary, this study enhanced the comprehension of the pathogenesis of UC and identified TIMP1 as a critical target in the ferroptosis associated with UC.

## Author Contributions

Xuan Zhou: project administration, resources, supervision, validation, writing—original draft. Qiao Qiao: formal analysis, software. Jie Yang: data curation, methodology. Huiming Tu: conceptualization, investigation, funding acquisition, visualization, writing—review and editing. Xuan Zhou and Qiao Qiao contribute equally to this study.

## Funding

No funding was received for this manuscript.

## Ethics Statement

The authors have nothing to report.

## Conflicts of Interest

The authors declare no conflicts of interest.

## Data Availability

The data that support the findings of this study are available from the corresponding author upon reasonable request.
